# Hippocampal Upregulation of Complement Component C3 in Response to Lipopolysaccharide Stimuli in a Model of Fragile-X Syndrome

**DOI:** 10.3390/cimb45110582

**Published:** 2023-11-18

**Authors:** Danielle Santana-Coelho, Joaquin N. Lugo

**Affiliations:** 1 Department of Psychology and Neuroscience, Baylor University, Waco, TX 76798, USA; coelhod@uthscsa.edu; 2 Institute of Biomedical Studies, Baylor University, Waco, TX 76798, USA; 3 Department of Biology, Baylor University, Waco, TX 76798, USA

**Keywords:** autism, autism spectrum disorder, complement, neuroinflammation, cytokines, FXS

## Abstract

The complement system is part of the innate immune system and has been shown to be altered in autism spectrum disorder (ASD). Fragile-X syndrome (FXS) is the main genetic cause of ASD and studies suggest a dysregulation in the immune system in patients with the disorder. To assess if an animal model of FXS presents with altered complement signaling, we treated male *Fmr1* knockout (KO) mice with lipopolysaccharide (LPS) and collected the hippocampus 24 h later. Assessment of the expression of the complement genes C1q, C3, and C4 identified the upregulation of C3 in both wild-type (WT) and knockout mice. Levels of C3 also increased in both genotypes. Analysis of the correlation between the expression of C3 and the cytokines IL-6, IL-1β, and TNF-α identified a different relationship between the expression of the genes in *Fmr1* KO when compared to WT mice. Our findings did not support our initial hypotheses that the lack of the *FMR1* gene would alter complement system signaling, and that the induction of the complement system in response to LPS in Fmr1 KO mice differed from wild-type conspecifics.

## 1. Introduction

Alterations in the immune system are associated with the pathophysiology of neurodevelopmental disorders [1]. Increased levels of cytokines have been found in individuals with autism spectrum disorder, suggesting that these immune-regulatory molecules may be involved in the pathophysiology of this developmental disorder [2,3,4,5,6]. In support of this hypothesis, preclinical studies have shown that an immune stimulus during the perinatal period can induce the development of an autistic-like phenotype in rodents and primates [7,8,9,10,11]. Furthermore, neutralization of certain cytokines, such as IL-6, can prevent the development of the autistic-like phenotype [12,13].

The complement system is a part of the innate immune system, consisting of a cascade of proteases that interact with one another resulting in the elimination of pathogens. In the past two decades, studies have demonstrated that the complement system is involved in synaptic pruning, an important developmental process that can be altered in developmental disorders. The proteins C1q and C3 tag synapses for elimination that are recognized by microglia through C3 receptors [14,15,16]. A growing body of evidence associates alterations in the complement system with ASD [17,18,19,20,21]. Warren and collaborators identified decreased levels of the complement protein C4b in autistic children and their mothers’ plasma [22]. A study by Fagan and collaborators showed that autistic individuals presented with the downregulation of C3 in the middle frontal gyrus when compared to typical individuals. Additionally, decreasing C3 in rodents’ prefrontal cortex induced the development of an autistic-like phenotype [18]. A study with iPSC-derived astrocytes from ASD patients showed reduced levels of C4 [21]. Together, these studies suggest that alterations in the complement system proteins C3 and C4 may be a translatable finding in ASD and a possible target for interventions.

FXS is the main single-gene cause of autism spectrum disorder (ASD), being responsible for 2–6% of ASD diagnoses [23]. The disorder is caused by an X chromosome mutation with abnormal expansion CGG repeats (>200 repeats). This mutation leads to the silencing of the transcription of the Fragile X Messenger Ribonucleoprotein 1 (*FMR1*) gene, decreasing the levels of the Fmr1 protein [23,24]. Some of the pathological findings in individuals with FXS and animal models of the disorder are an altered dendritic spine morphology [25,26] and long-term depression [27]. The role of the immune system in FXS has also been previously investigated. A study showed that the level of proinflammatory chemokines was reduced in the serum of FXS individuals [28]. Additionally, CGG repeats are associated with immune dysregulation [29]. Careaga and collaborators found that human premutation carriers and *FMR1* knock-in mice presented decreased levels of cytokines in the periphery when stimulated with lipopolysaccharide [29]. These findings show that FXS is accompanied by alterations in the immune system.

To date, the status of the complement system signaling has not been extensively investigated in Fragile-X syndrome. We hypothesize that the lack of the *FMR1* gene may alter the complement system signaling since clinical and preclinical data suggest an altered immune phenotype when the Fmr1 protein expression is decreased. Therefore, we used *Fmr1* KO mice and administered LPS to induce an acute immune response. This experimental approach enabled us to assess not only if the lack of Fmr1 protein can cause baseline alterations in the complement system, but also if the induction of the complement system in response to LPS in *Fmr1* KO mice differs from that in wild-type conspecifics.

## 2. Materials and Methods

### 2.1. Animals

Experimental subjects included adult male *Fmr1* knockout and wild-type mice on a C57BL/6J background strain (Jackson Laboratory). Breeding pairs consisted of male WT sire and *Fmr1* Het dams. Mice were bred and housed at Baylor University in standard laboratory conditions (22 °C, 12 h light/dark cycle) and had *ad libitum* access to food and water throughout the experiment. All procedures were performed in compliance with the Baylor University Institutional Animal Care and Use Committee and the *National Institute of Health Guidelines for the Care and Use of Laboratory Animals.*

### 2.2. Lipopolysaccharide Stimulation

The bacterial endotoxin lipopolysaccharide (LPS) was administered in a single intraperitoneal (i.p.) injection of 0.83 mg/kg LPS from *Escherichia coli* serotype O127:B8 purified by gel-filtration chromatography (Sigma, St. Louis, MO, USA). Control mice were treated with an equivalent volume of 0.9% physiological saline. Twenty-four hours after LPS stimulation, the animals were anesthetized with isoflurane, blood was collected by cardiac puncture, and the mice were perfused with ice-cold PBS. Hippocampi were rapidly dissected and frozen in dry ice. Blood samples were centrifuged at 1500× *g* at 4 °C for 10 min. Plasma was collected and frozen in dry ice. Samples were maintained at −80 °C until processing.

### 2.3. RNA Isolation and RT-qPCR

One hippocampus was homogenized in 300 μL of lysis buffer, and total RNA was isolated according to the manufacturer’s instructions using the RNeasy Mini Kit (Qiagen, Hilden, Germany). RNA concentration was measured using a NanoDrop ND-1000 Spectrophotometer (Thermo Scientific, NanoDrop Products, Wilmington, DE, USA). Expression levels of the complement pathway and cytokine genes were determined by quantitative reverse transcription-polymerase chain reaction (qRT-PCR). A Reverse Transcription Kit was used to produce complementary DNA (cDNA), according to the manufacturer’s instructions (Applied Biosystems, Carlsbad, CA, USA). Taqman probes and primer were used to determine the expression of hippocampal mRNA by quantitative RT-PCR on a QuantStudio 6 Real-Time PCR System (Applied Biosystems, Carlsbad, CA, USA). Relative gene expression levels were determined for TNF-α (Mm00443258_m1), IL-6 (Mm00446190_m1), C3 (Mm01232779_m1), C1q (Mm00482904_m1), C4 (Mm00432150_m1) by normalizing gene expression to the housekeeping gene β-actin (Mm00607939_s1). Data are expressed as fold change relative to WT saline controls, which was calculated using the formula 2^−ΔCt^, where ΔCt represents the magnitude of the difference between target and endogenous cycle threshold (Ct) values.

### 2.4. Western Blotting

Western blotting was used to further investigate the upregulation of the complement protein C3. Briefly, the hippocampus was homogenized, and total homogenates were used for Western blotting analysis. The primary antibodies used in the study were anti-C3 (1:500, ab200999, Abcam, Cambridge, UK) and anti-Actin (1:1000, A2066, Sigma). Western blotting procedures were run as previously described [30].

### 2.5. ELISA

Plasma samples were thawed, and IL-6 was measured using a commercially available mouse IL-6 Uncoated ELISA Kit (Invitrogen, Waltham, MA, USA), following the manufacturer’s protocol. Data are expressed in pg/mL.

### 2.6. Statistical Methods

Statistical analyses were performed using the GraphPad Prism 7 software (La Jolla, CA, USA) and SPSS28 (IBM, Chicago, IL, USA). A two-way repeated-measures ANOVA, with a within-subjects factor of time (baseline, 24 h) and between-subjects factors of treatment (Saline, LPS) and genotype (WT, *Fmr1* KO), was used to analyze changes in weight over time following LPS administration. A two-way ANOVA was used to analyze changes in IL-6 plasma levels and the expression of hippocampal cytokines and complement system proteins in response to LPS. Pearson correlation was used to assess the relationship between cytokines and C3 expression using ΔCT values. A value of *p* < 0.05 was considered significant for all analyses. Data in the figures are expressed as the mean ± standard error of the mean (SEM).

## 3. Results

### 3.1. The Peripheral Immune Response to LPS Is Similar between Wild-Type and Fmr1 KO Mice

**Figure 1:**
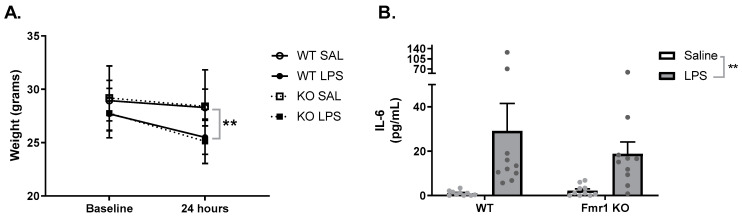
Response to immune stimuli is similar in both genotypes. (**A**) Weight was measured at baseline and 24 h after LPS administration. LPS decreased the weight of the mice in both genotypes in a similar manner. WT SAL N = 10, WT LPS N = 10, KO SAL N = 10, KO LPS = 9. (**B**) Levels of IL-6 were measured 24 h after LPS treatment. LPS increased the levels of IL-6 in both genotypes in a similar manner. WT SAL N = 10, WT LPS N = 10, KO SAL N = 10, KO LPS = 9. Data are expressed as mean ± SEM. Each dot represents a different mouse. Weight data were analyzed with a two-way repeated-measures ANOVA. IL-6 levels were analyzed with two-way ANOVA. ** < 0.01.

To confirm that the mice in the study had a sickness response to LPS, we measured their weight before the administration of LPS (baseline) and 24 h after LPS. Two-way repeated-measures ANOVA identified a within-subjects interaction between weight and treatment (*F*_1,35_ = 47.49, *p* < 0.001, Figure 1A). *Post hoc* analysis revealed that, 24 h after the administration of LPS, the treated group presented a significant decrease in weight when compared to controls (*p* < 0.001). To verify if the immune response to LPS was altered in *Fmr1* KO mice, we measured the peripheral levels of the proinflammatory cytokine IL-6 24 h after LPS administration. Two-way ANOVA revealed a main effect of treatment (*F*_1,36_ = 10.85, *p* < 0.01, Figure 1B), where LPS induced a significant increase in the levels of IL-6 in both genotypes. No main effect of genotype (*F*_1,36_ = 0.45, *p* = 0.50) or the interaction (*F*_1,36_ = 0.72, *p* = 0.39) between genotype and treatment was identified.

### 3.2. The Central Nervous System Immune Response to LPS Is Similar between Wild-Type and Fmr1 KO Mice

**Figure 2:**
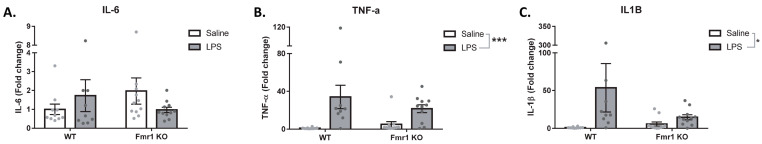
LPS induces TNF-α and IL-1β expression independent of the genotype. Expression of the cytokines IL-6 (**A**), TNF-α (**B**), and IL-1β (**C**) was measured 24 h after LPS treatment. WT SAL N = 10, WT LPS N = 9, KO SAL N = 11, KO LPS = 11. Data are expressed as mean ± SEM and were analyzed with two-Way ANOVA. * < 0.05 and *** < 0.001. Each dot represents a different mouse.

To assess if *Fmr1* KO mice had an altered central nervous system response to LPS, we measured the expression of the cytokines IL-6, TNF-α, and IL-1β in hippocampal homogenates. Two-way ANOVA revealed no significant alterations in the levels of IL-6 in any of the treated groups 24 h after LPS administration (Figure 2A). A main effect of treatment showed that LPS increased the levels of the cytokines TNF-α (*F*_1,37_ = 16.65, *p* = 0.002) and IL-1β (*F*_1,37_ = 4.60, *p* = 0.03), independent of the genotype (Figure 2B,C). No main effects of genotype (TNF-α: *F*_1,37_ = 0.47, *p* = 0.49; IL-1β: *F*_1,37_ = 1.51, *p* = 0.22) or the interaction (TNF-α: *F*_1,37_ = 1.81, *p* = 0.18; IL-1β: *F*_1,37_ = 2.42, *p* = 0.12) between genotype and treatment were identified for any of the cytokines.

### 3.3. C3 Is Upregulated in LPS-Treated Mice Independent of the Genotype

**Figure 3:**
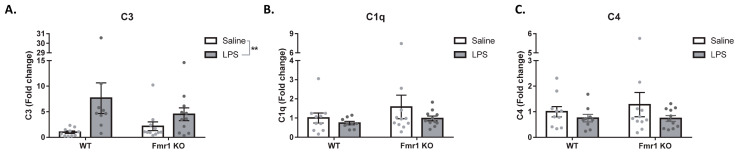
The expression of C3 is upregulated 24 h after LPS. Expression of the complement C3 (**A**), C1q (**B**), and C4 (**C**) was measured 24 h after LPS treatment. WT SAL N = 10, WT LPS N = 9, KO SAL N = 11, KO LPS = 11. Data are expressed as mean ± SEM and were analyzed with two-way ANOVA. ** < 0.01. Each dot represents a different mouse.

The expression of the complement system proteins C3, C1q, and C4 was measured in the hippocampus to investigate if *Fmr1* KO mice responded differently than WT to the effects of LPS on the complement system. A main effect of treatment was identified for C3, where the expression of C3 was upregulated by the LPS treatment (*F*_1,37_ = 8.446, *p* = 0.0061, Figure 3A). No main effect of genotype (*F*_1,37_ = 0.40, *p* = 0.52) or the interaction (*F*_1,37_ = 1.90, *p* = 0.17) was identified. Regarding C1q and C4, no significant main effect of treatment (C1q: *F*_1,37_ = 1.57, *p* = 0.21; C4: *F*_1,37_ = 1.85, *p* = 0.18), genotype (C1q: *F*_1,37_ = 1.42, *p* = 0.24; C4: *F*_1,37_ = 0.19, *p* = 0.66), or their interaction (C1q: *F*_1,37_ = 0.31, *p* = 0.57; C4: *F*_1,37_ = 0.26, *p* = 0.61) was identified.

**Figure 4:**
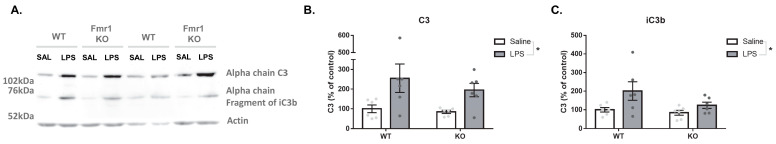
C3 levels are increased by LPS independent of the genotype. (**A**) Representative Western blotting with antibodies for C3 fragment (alpha chain C3, ~115 kDa, alpha chain fragment of iC3b, ~68 kDa) and Actin (loading control, 42 kDa). Level of C3 fragments was measured in the hippocampus 24 h after LPS administration. Alpha chain of C3 (**B**) and iC3b fragment (**C**) were increased in response to LPS in both genotypes. WT SAL N = 6, WT LPS N = 6, KO SAL N = 6, KO LPS = 6. Data are expressed as mean ± SEM and were analyzed with two-way ANOVA. * < 0.05. Each dot represents a different mouse.

To confirm the upregulation identified for C3 with qPCR, we measured the levels of the protein C3 using Western blotting. The classical complement pathway induces the cleavage of the protein C3. C3 is a protein formed by an alpha (α) and a beta chain (β). C3α is cleaved into C3a and C3b by C3 convertase. C3b is cleaved into iC3b, which is an opsonin associated with the phagocytosis of targeted cells [31]. Here, we were able to measure the alpha chain of C3 (~115 kDa) and the fragment iC3b (~68 kDa, Figure 4A). Two-way ANOVA identified a main effect of treatment for alpha chain C3 (*F*_1,20_ = 5.85, *p* = 0.02, Figure 1B) and iC3b (*F*_1,20_ = 6.39, *p* = 0.02, Figure 1C), suggesting that LPS increased the levels of the proteins in a similar manner in both genotypes.

### 3.4. The Correlation between the Expression of Cytokines and C3 Is Dependent on Genotype at Baseline

**Figure 5:**
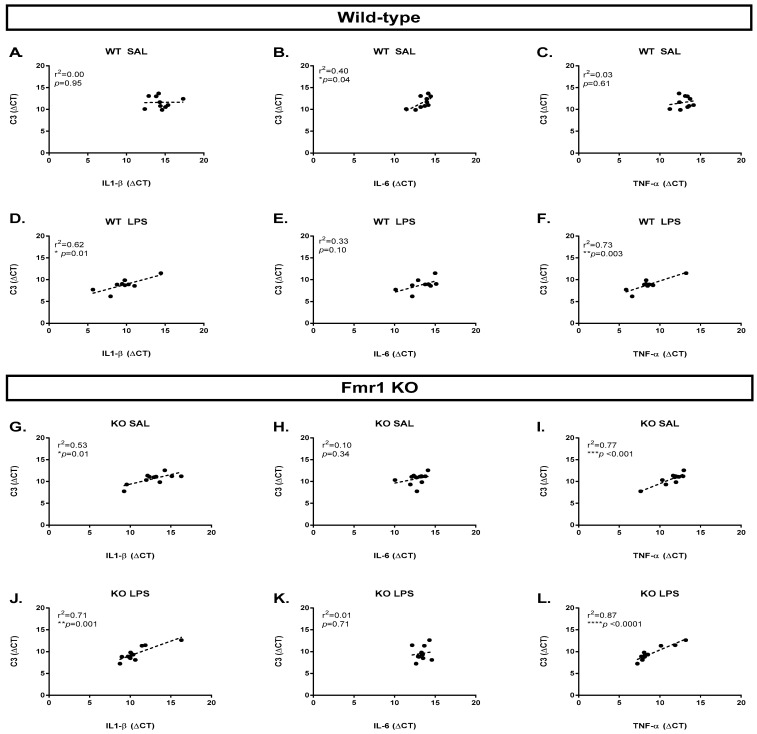
Correlation between cytokines and C3 is dependent on genotype at baseline. Pearson correlation was performed between the expression of C3 and the cytokines IL-1β, IL-6, and TNF-α. WT SAL N = 10, WT LPS N = 9, KO SAL N = 11, KO LPS = 10. (**A**) Correlation between C3 and IL-1β in WT SAL group. (**B**) Correlation between C3 and IL-6 in WT SAL group. (**C**) Correlation between C3 and TNF-α in WT SAL group. (**D**) Correlation between C3 and IL-1β in WT LPS group. (**E**) Correlation between C3 and IL-6 in WT LPS group. (**F**) Correlation between C3 and TNF-α in WT LPS group. (**G**) Correlation between C3 and IL-1β in *Fmr1* KO SAL group. (**H**) Correlation between C3 and IL-6 in *Fmr1* KO SAL group. (**I**) Correlation between C3 and TNF-α in *Fmr1* KO SAL group. (**J**) Correlation between C3 and IL-1β in *Fmr1* KO LPS group. (**K**) Correlation between C3 and IL-6 in *Fmr1* KO LPS group. (**L**) Correlation between C3 and TNF-α in *Fmr1* KO LPS group. Gene expression is expressed as ΔCT. * *p* < 0.05. *p* ** < 0.01, *p* *** < 0.001, and **** *p* < 0.0001.

LPS-induced C3 expression in microglia and astrocytes can be mediated by TNF-α [32]. To assess if a similar relationship between a cytokine and C3 could be altered in *Fmr1* KO mice, we determined the correlation between the expression of C3 and the cytokines that we measured in the study. Pearson correlation indicated that, at baseline, IL-6 was correlated with the expression of C3 in wild-type mice (r^2^ = 0.40, *p* = 0.04, Figure 5B), while the same was not true for *Fmr1* KO mice (Figure 5H). Alternatively, IL1-β (r^2^ = 0.53, *p* = 0.01, Figure 5G) and TNF-α (r^2^ = 0.77, *p* < 0.001, Figure 5I) were correlated with C3 expression in *Fmr1* KO at baseline. Twenty-four hours after LPS administration, C3 expression was correlated with IL-1β (WT LPS: r^2^ = 0.62, *p* = 0.01, Figure 5D; KO LPS: r^2^ = 0.71, *p* = 0.001, Figure 5J) and TNF-α (WT LPS: r^2^ = 0.73, *p* = 0.003, Figure 5F; KO LPS: r^2^ = 0.87, *p* < 0.0001, Figure 5L) in both genotypes.

## 4. Discussion

The role of the immune system in the pathogenesis of developmental disorders has been demonstrated in the past few years. Recent findings suggest that the complement system is also altered in developmental disorders such as ASD [19,21]. Fragile-X syndrome is a developmental disorder that can be part of the autism spectrum, and previous studies have identified, in both humans and rodents, that the deficiency of the *Fmr1* protein can be accompanied by an altered immune response [29]. However, the complement system has not been previously assessed in *Fmr1* mutants on the C57BL/6 background. Hence, we aimed to investigate if *Fmr1* mutants present altered complement system function at baseline and in response to an immune stimulus. Here, we show that LPS administration causes an inflammatory response characterized by the increased expression of cytokines and C3 in hippocampi homogenates in both WT and *Fmr1* knockout mice.

LPS is known to decrease food consumption and weight in the first 24 h after administration [33,34]. Additionally, cytokine levels increase in the periphery and central nervous system [33,35]. Here, we confirmed these effects of LPS by showing a weight loss and an increase in the plasma level of IL-6 in the treated animals when compared to controls. To further characterize the immune response, we measured the expression of the proinflammatory cytokines IL-6, TNF-α, and IL-1β in hippocampal homogenates. The expression of TNF-α and IL-1β was increased in the treated animals twenty-four hours after LPS administration. IL-6, however, was not found to have increased at this timepoint. IL-6 expression after LPS administration may have peaked at an earlier time and returned to lower levels by 24 h, similar to previous studies [36,37]. This early response could be responsible for the lack of upregulation at the 24 h timepoint [38]. Contrary to our hypothesis, no significant differences in the levels or expression of cytokines were identified between wild-type and *Fmr1* KO mice. A previous study from our laboratory identified an exaggerated immune response to LPS 4 h after the stimulus. Hodges and collaborators showed that 0.33 mg/kg increased the expression of IL-1β, TNF-α, MCP1, IL-6, and IL-10 in both wild-type and *Fmr1* KO. However, the upregulation of IL-1β and IL-6 was more robust in knockout mice when compared to wild-type mice, while the expression of TNF-α, MCP1, and IL-10 was similar between genotypes [33]. Yuskaitis and collaborators found no differences between the wild-type and *Fmr1* KO levels of TNF-α and IFN-γ four hours after the administration of 10 mg/kg LPS [39]. This study used the same background strain used in our study (C57BL6J) but a much higher dose of LPS. The same study also demonstrated the similar production of IL-6 and TNF-α by microglia in both genotypes when stimulated with LPS. In the present study, we demonstrate a brief snapshot of the immune response in these mice. A time course study might be necessary to further characterize if, at an earlier or later timepoint, these animals could present an altered immune response. Additionally, investigating whether there are alterations in anti-inflammatory cytokines would give us a more complete picture of the immune response in these mice.

To assess if the complement system was altered in *Fmr1* Kos, we measured the expression of the complement genes C3, C1q, and C4. We also assessed the mice’s complement response 24 h after stimulation with LPS to identify any alteration in the response to immune stimuli. We chose the 24 h timepoint based on previous studies that have demonstrated that C3 levels and expression are increased at 8 h [40], 24 h [41], and 5 days [42] after LPS administration. Here, we identified the upregulation of C3 in both genotypes, and no alterations in the expression of C1q and C4. We also measured the levels of C3 (~115 kDa) and the biologically active iC3b fragment (~75kDa) and found higher levels in LPS-treated mice independent of the genotype. C3 and iC3b are complement proteins that participate in the pruning of neurons during development in certain brain areas. These proteins label synapses for elimination and recognition by microglia, leading to the phagocytosis of the labelled spines [43]. Alterations in the complement system have been linked to autism and Fragile-X syndrome. Fagan and collaborators demonstrated that C3 was decreased in autistic subjects and the lack of C3 during development caused behavior deficits in rodents [18]. A recent study showed that *Fmr1* KO in the FVB background presented increased levels of C3 and C1q in the prefrontal cortex, which corroborated our initial hypothesis that *Fmr1* KO mice would present an altered complement system at baseline and potentially in response to inflammatory stimuli [44]. However, our data did not produce evidence that this was the case. The findings of the Fagan and Gao studies suggest that perhaps a broader analysis in additional brain areas other than the hippocampus might identify alterations in the complement system in *Fmr1* KO mice, considering that, in the current study, we assessed only hippocampal samples.

To further investigate if the immune response in the *Fmr1* KO is different than in wild types, we assessed the correlations between the expression of cytokines and the complement system target C3. The cytokines IL-6, IL-1β, and TNF-α have been previously shown to modulate C3 signaling [32,45]. In wild-type mice, we found a correlation between IL-6 and C3 at baseline, while, after LPS administration, C3 expression presented a positive correlation with the expression of IL-1β and TNF-α. In *Fmr1* KOs, we identified a correlation between C3 and the cytokines L-1β and TNF-α at baseline, and after LPS administration. IL-6, however, did not correlate with C3 in knockout mice. Wild-type mice appeared to have an increased response to LPS when comparing the correlations at baseline to after LPS administration. Meanwhile, *Fmr1* KOs appeared to have an altered relationship between the targets at baseline and a milder response to the immune stimuli when comparing baseline to after LPS correlations. These differences between wild types and knockouts suggest that the relationship between the cytokines and C3 may be altered in *Fmr1* KOs. However, further investigation of this relationship with the direct administration of these cytokines is necessary to confirm the correlations.

## 5. Conclusions

Together, our data indicate that *Fmr1* KOs’ complement system responds similarly to that of wild-type mice 24 h after LPS administration. However, the correlation analysis suggests that the relationship between cytokines and C3 expression may be altered in knockouts. To our knowledge, this is one of the first studies to investigate the state of the complement system in an animal model of Fragile-X syndrome and the first to do so in the C57BL6 background strain. More studies are necessary to assess if, at different timepoints, doses, and brain regions, the lack of Fmr1 protein alters complement signaling.

## Data Availability

We are willing to provide all data upon request. Please contact the corresponding author.
